# Improving Artificial Photosynthesis over Carbon Nitride by Gas–Liquid–Solid Interface Management for Full Light‐Induced CO_2_ Reduction to C_1_ and C_2_ Fuels and O_2_

**DOI:** 10.1002/cssc.201903515

**Published:** 2020-02-11

**Authors:** Yang Xia, Kai Xiao, Bei Cheng, Jiaguo Yu, Lei Jiang, Markus Antonietti, Shaowen Cao

**Affiliations:** ^1^ State Key Laboratory of Advanced Technology for Materials Synthesis and Processing Wuhan University of Technology Wuhan 430070 P. R. China; ^2^ Department of Colloid Chemistry Max Planck Institute of Colloids and Interfaces 14476 Potsdam Germany; ^3^ Key Laboratory of Bio-inspired Smart Interfacial Science and Technology of Ministry of Education School of Chemistry Beihang University 100191 Beijing P. R. China

**Keywords:** carbon nitride, CO_2_ reduction, interfaces, photocatalysis, solar fuels

## Abstract

The activity and selectivity of simple photocatalysts for CO_2_ reduction remain limited by the insufficient photophysics of the catalysts, as well as the low solubility and slow mass transport of gas molecules in/through aqueous solution. In this study, these limitations are overcome by constructing a triphasic photocatalytic system, in which polymeric carbon nitride (CN) is immobilized onto a hydrophobic substrate, and the photocatalytic reduction reaction occurs at a gas–liquid–solid (CO_2_–water–catalyst) triple interface. CN anchored onto the surface of a hydrophobic substrate exhibits an approximately 7.2‐fold enhancement in total CO_2_ conversion, with a rate of 415.50 μmol m^−2^ h^−1^ under simulated solar light irradiation. This value corresponds to an overall photosynthetic efficiency for full water–CO_2_ conversion of 0.33 %, which is very close to biological systems. A remarkable enhancement of direct C2 hydrocarbon production and a high CO_2_ conversion selectivity of 97.7 % are observed. Going from water oxidation to phosphate oxidation, the quantum yield is increased to 1.28 %.

## Graphical abstract

**Access all areas**: A triphase interfacial CO_2_ reduction system over a carbon nitride photocatalyst enables the reactant CO_2_ to reach the reaction interface directly from the ambient atmosphere, which simultaneously enhances the reaction rate and suppresses the competitive hydrogen evolution.

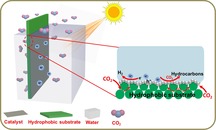

Ever‐increasing consumption of fossil fuels along with the massive emission of carbon dioxide (CO_2_) has generated an energy crisis and resulted in climate change.^1^ Artificial photosynthesis through photocatalytic CO_2_ conversion into valuable chemicals (e.g., CO or H_2_, and, preferably, CH_4_, C_2_H_4_, etc.) in the presence of H_2_O has been recognized as a potentially promising way to resolve these issues.^2^ The transfer of photogenerated charge carriers and mass transport play crucial roles in determining the kinetics of catalysts and CO_2_ photoreduction efficiency.^3^ Meanwhile, the competitive reaction of photocatalytic hydrogen evolution also diminishes the generation of hydrocarbons, resulting in low selectivity and activity of CO_2_ reduction of most current systems. To overcome kinetic limitations and suppress the hydrogen evolution reaction, numerous efforts have focused on the improvement of pristine photocatalysts, by methods such as loading cocatalysts,^4^ tailoring morphologies,^5^ adjusting defect densities,^6^ and constructing heterojunctions.^7^ At the same time, the reaction interface that governs the solid–liquid contact and mass transfer is also of vital importance to the photocatalytic CO_2_ conversion process. Previous studies found that the availability of excess protons (H^+^) and low concentration of CO_2_ at the reaction interface lead to unsatisfactory activity and selectivity of the photocatalytic CO_2_ reduction system.^8^ In a conventional liquid–solid diphase system for CO_2_ photoreduction, the availability of CO_2_ at the reaction interface is dependent on its mass transfer through the water phase.^9^ The low concentration and slow diffusion rate of CO_2_ molecules in water thereby strongly hinder the surface catalytic process of CO_2_ photoreduction.

In this study, to overcome the limitations of the conventional liquid–solid diphase system of CO_2_ photoreduction, a simple and sustainable approach is developed by constructing a triphase (gas–liquid–solid) interfacial photocatalytic system. The photocatalysts are immobilized on the surface of a carbon fiber substrate (Figure 1). The concentration of CO_2_ molecules at the interface can be controlled by adjusting the surface adsorption on the substrate. Particularly, a hydrophobic substrate surface promotes CO_2_ localization from the gas phase and helps to rapidly deliver CO_2_ molecules to the contact area of gas (CO_2_), liquid (water), and solid (catalyst). Such a reaction system then allows the continuous delivery of CO_2_ molecules from the gas phase to the reaction interface via its hydrophobic channels, instead of the slow diffusion through the liquid phase. As a result, the accessibility of CO_2_ molecules to the photocatalyst is greatly increased, which subsequently enhances the rate of the reaction between CO_2_ and photogenerated electrons, thereby diminishing electron–hole recombination and increasing charge utilization. Finally, the activity and selectivity of photocatalytic CO_2_ conversion is remarkably improved.

**Figure 1:**
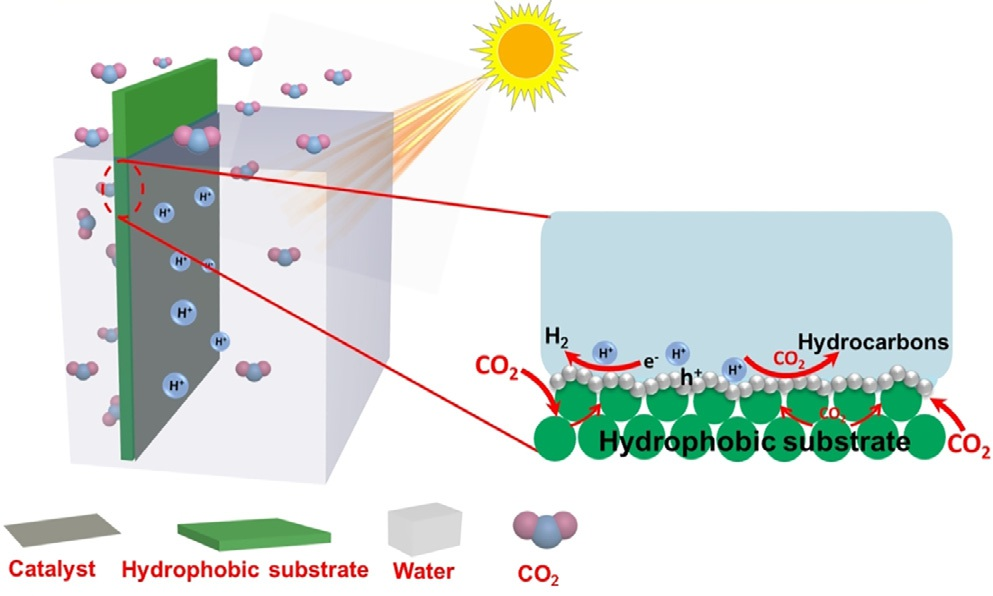
Schematic illustration of the triphase photocatalytic system with enlarged view of the solid–liquid–air triphase reaction interface (right).

As a proof of concept, we immobilized carbon nitride (CN) nanosheets onto the surface of a carbon fiber (CF) fleece with porous structure and different wettability. The contact angles (CAs) of the superhydrophilic, hydrophilic, and hydrophobic CF substrates are approximately 0°, 37.5°, and 112°, respectively (Figure 2 A–C). After CN immobilization on one side of the substrate, the side with the CN layer becomes superhydrophilic or hydrophilic with CAs of approximately 0°, 0° and 10° (see the Supporting Information, Figure S1; the corresponding samples are denoted as CN/CF1, CN/CF2, and CN/CF3, respectively). In this case, water can wet the hydrophilic photocatalyst layer while gas‐phase CO_2_ is directed through the hydrophobic substrate up to the CN particles, resulting in the formation of a gas–liquid–solid triphase boundary zone. Such a framework then secures the supply of both abundant water and CO_2_ molecules to drive overall CO_2_ photoreduction. Field‐emission scanning electron microscopy (FESEM) allows direct observation of the interface between immobilized CN photocatalysts and carbon fiber substrates (Figure 2 D, E).

**Figure 2:**
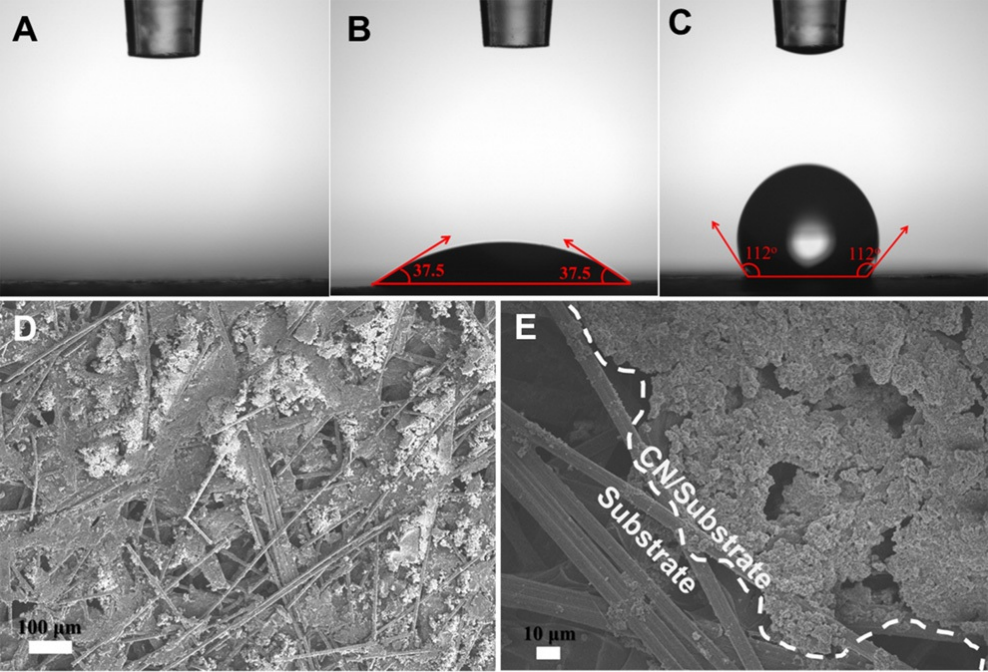
Contact angle measurement of water on (A) superhydrophilic CF, (B) hydrophilic CF, and (C) hydrophobic CF. FESEM images of CN coating on the surface of hydrophobic porous CF at (D) low and (E) high magnifications.

The powder X‐ray diffraction (XRD) patterns of CF, CN/CF1, CN/CF2, CN/CF3, and CN are shown in Figure 3 A. The pattern of CF exhibits a broad peak at around 26.5°, indexed as the (002) plane of a graphitic carbon structure, the fleece.^10^ Two distinct diffraction peaks appeared at 13.1° and 27.3°, corresponding to the (100) and (002) planes of CN, respectively. The former corresponds to the repeated structural packing of tri‐*s*‐triazine heterocycles in the conjugated planes, and the latter can be ascribed to the regular graphite‐like interlayer stacking.^11^ After the immobilization of CN onto the surface of the carbon fiber substrate, the XRD patterns of CN/CF show the characteristic peaks of both CN and CF. The peak intensity of CF in CN/CF samples gradually decreases from CN/CF1 to CN/CF3. This is due to the different surface energy of CF with varying surface wettability, resulting in different film thicknesses of the CN layer,^12^ although the primary loading of CN is identical. The optical absorption properties of all samples were then measured by UV/Vis diffuse reflectance spectroscopy (DRS; Figure 3 B). For CN, the absorption edge at 450 nm corresponds to its intrinsic band gap of 2.76 eV. CN/CF1, CN/CF2, and CN/CF3 show similar absorption edges but enhanced light‐absorption intensity in visible‐light regions, owing to the strong broad absorption of CF.^13^ Particularly, the absorption in this region of hydrophilic CF‐supported CN coated is stronger than that of hydrophobic CF‐supported CN, because of the different film thickness of photocatalyst layer, consistent with the XRD results. The different thicknesses of CN films affected the scattering of light among the texture and pore structure in CF substrates, which led to differences in the light absorption in the visible‐light region over CN/CF samples. FT‐IR spectroscopy was used to further investigate the surface structure of CN and CN/CF samples (Figure 3 C). The broad peaks between 3000 and 3500 cm^−1^ can be ascribed to the adsorbed hydroxy groups and the amino groups in CN, whereas the peaks at around 803, 1211, 1402, 1531, and 1639 cm^−1^ are the typical stretching vibrations of the *s*‐triazine ring system, C=N and C‐N heterocycles, respectively.^14^ All CN/CF samples show the same characteristic peaks as those of CN, suggesting that the coating of CN onto the surface of CF has no obvious effect on the structure of CN, and that CF only serves a platform for the immobilization of CN. In order to reveal the effect of surface wettability of CF on the charge separation efficiency for CN, photoluminescence (PL) spectra were measured (Figure 3 D). The intensity of all PL spectra is similar, indicating that the charge recombination rate in the samples of CN immobilized on CF with different surface wettability is similar. Namely, the expected performance difference of photocatalytic CO_2_ reduction could not be related to variation in the charge transfer dynamics within the series of catalysts.

**Figure 3:**
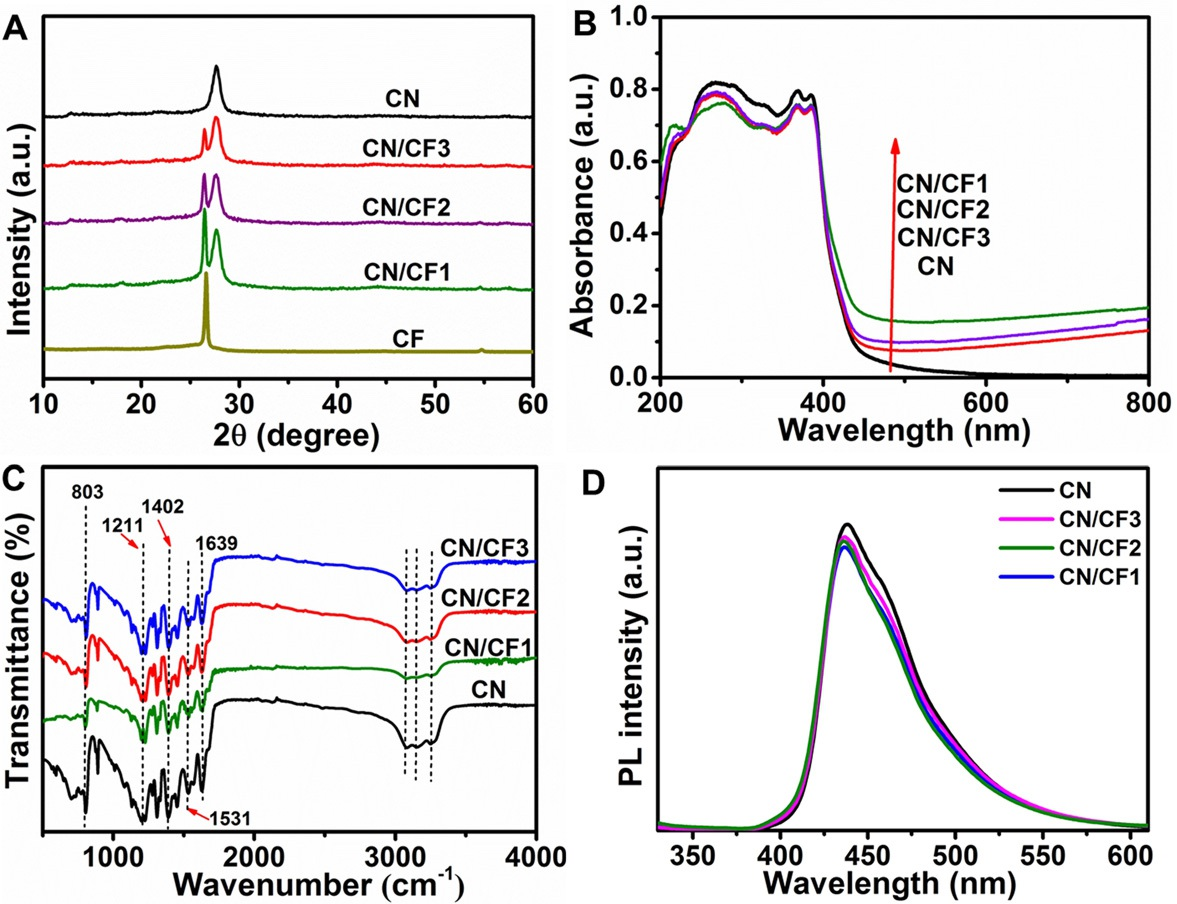
(A) XRD patterns, (B) UV/Vis DRS, (C) FTIR spectra, and (D) PL spectra of various samples.

The photocatalytic activity and selectivity of as‐prepared CN/CF photocatalysts were investigated under simulated sunlight irradiation in the absence of any sacrificial agent and co‐catalyst. The novel reaction environment of gas–liquid–solid (G–l–S) triphase reaction interface was employed (Figure S2). As a result, a spectrum of product molecules is found (Figure 4 A and Table S1); the main products of CO_2_ reduction are CH_4_, CO, C_2_H_4_ and H_2_ over CN/CF1, CN/CF2, and CN/CF3. On increasing the hydrophobicity of the CF substrate, the generation of hydrocarbon fuels from CO_2_ reduction reaction massively increases, whereas that of H_2_ from water splitting is suppressed. In particular, CN/CF3 shows the best performance with a total CO_2_ conversion of 415.50 μmol m^−2^ h^−1^, and a corresponding quantum yield of 0.33 %, which is higher than or comparable to reported results.^15^ This is of the order of natural photosynthesis, albeit here described with a much simpler synthetic system, free of further cocatalysts to increase the rate of hydrocarbon formation, as well as oxygen liberation. The CO_2_ conversion selectivity is as high as 97.7 %, as compared to that of H_2_ evolution. that is, 97.7 % of all electrons end up in carbon products. The rate of generation of C2 hydrocarbon product (C_2_H_4_), as well as that of CH_4_, over the hydrophobic substrate is significantly higher than that over the hydrophilic substrate. It can be concluded that CN immobilized onto the hydrophobic substrate experiences a continuous supply of CO_2_ molecules, thus making CO_2_ reduction processes much more effective than H_2_ generation under the given conditions of no metal cocatalyst. Moreover, increasing the loading amount of CN would further enhance the photocatalytic activity while maintain the high selectivity (see data of CN/CF4 in Table S1).

**Figure 4:**
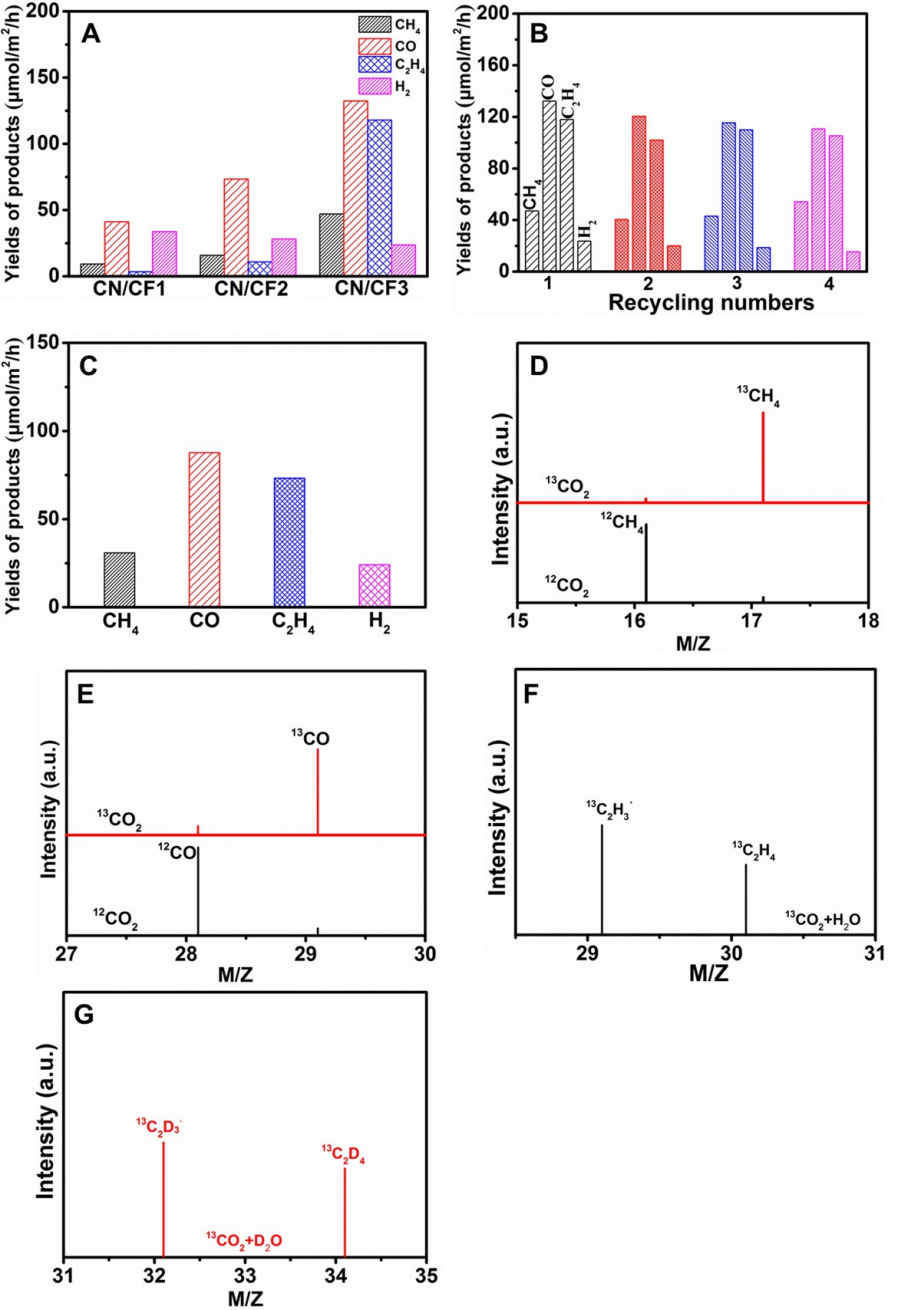
A) Photocatalytic activity of CN/CF1, CN/CF2, and CN/CF3 in the triphase system with CO_2_ atmosphere connection. B) Cycling test of photocatalytic CO_2_ reduction over CN/CF3 in the triphase system. C) Photocatalytic activity of CN/CF3 in the diphase system completely immersed in the water. D, E) GC‐MS analysis of reaction products CH_4_ (D) and CO (E) over CN/CF3 in the triphase system after irradiation for several hours with ^12^C and ^13^C as carbon sources. F, G) GC‐MS analysis of reaction products ^13^C_2_H_4_ (F) and ^13^C_2_D_4_ (G) over CN/CF3 in the triphase system after irradiation for several hours with ^13^CO_2_ as carbon source and D_2_O and H_2_O as hydrogen sources.

Furthermore, the good photocatalytic stability of CN/CF3 was demonstrated by a cycling test (Figure 4 B). The XRD pattern and contact angle of CN/CF3 were measured after the cycling test and showed no obvious change (Figure S3). To understand the unique interfacial effect, a controlled experiment was conducted in which the hydrophobic substrate immobilizing with CN (CN/CF3) was completely immersed in the water, which is similar with the conventional liquid–solid diphasic system (Figure S4). In this case, there was no direct contact between the substrate and CO_2_ atmosphere. Here, the necessary CO_2_ could only be supplied from the liquid phase. This test was conducted under the same reaction conditions as the triphase system. The main products of CO_2_ reduction in this liquid–solid diphase system are CH_4_, CO, C_2_H_4_ and H_2_, respectively. The total CO_2_ conversion reached 265.16 μmol m^−2^ h^−1^ (Figure 4 C). Indeed, by comparing the two experiments, we find the CO_2_ conversion rate in the triphase system to be twice that in the diphase system.

In addition, the production of O_2_ from water oxidation is the sole reaction to consume the photogenerated holes. Thus, we also monitored O_2_ evolution, which occurred at a rate of 711.95 μmol m^−2^ h^−1^ (Figure S5) by CO_2_ photoreduction over CN/CF3 in the triphase system, which is higher than the stoichiometric yield calculated from the given product distribution (644.34 μmol m^−2^ h^−1^). This deviation is reasonable within experimental error and might be related to undetectable species, such as methanol and ethanol, which end up dissolved in the water phase.

To confirm the carbon source of the photocatalytic products, isotope‐labeled CO_2_ was employed, and the hydrocarbon products carrying the isotopes ^13^C, ^12^C, and ^2^H (D) were detected by GC‐MS for the triphase system (Figure 4 D–G and Figure S6). The products labeled by ^12^C appear at earlier retention times than those labeled by ^13^C (Figure S6A). Clearly, the signals *m*/*z*=16.1 (^12^CH_4_) and *m*/*z=*17.1 (^13^CH_4_) were dominant in the GC‐MS spectra for photocatalytic ^12^C‐labeled CO_2_ and ^13^C‐labeled CO_2_ reduction, respectively (Figure 4 D). The signal at *m*/*z=*28.1 can be attributed to ^12^CO and ^12^C_2_H_4_, and that at *m*/*z=*29.1 can be attributed to ^13^CO when using ^12^CO_2_ and ^13^CO_2_, respectively (Figure 4 E). Subsequently, the isotope‐labeling experiments with carbon and hydrogen sources labeled with ^13^C and ^2^H (D) were performed for photocatalytic CO_2_ reduction over CN/CF3 (Figure S6B). The signals for molecular ethylene are at *m*/*z=*30.1 and *m*/*z=*34.1 when ^13^CO_2_ reacts with H_2_O and D_2_O, respectively. The signals at *m*/*z=*29.1 and *m*/*z=*32.1 are stronger those at *m*/*z=*30.1 and *m*/*z=*34.1, owing to the higher stability of the molecular ions (Figure 4 F, G).^16^ In conclusion, these results strongly indicate that the photocatalytic products originate solely from CO_2_ reduction.

In light of the above analysis, it is reasonable to propose a model to analyze the excellent CO_2_ photoreduction performance of CN/CF samples in the triphase reaction system (Figure 5 A, B). When the hydrophilic substrate was used to anchor the CN catalyst, a tiny proportion of CO_2_ molecules is supplied directly from the gas phase, and the majority is supplied from liquid phase to participate in photocatalytic CO_2_ reduction. Thus, the CO_2_ consumption and supply is unbalanced, resulting in a lower CO_2_ conversion rate. In contrast, when the hydrophobic substrate was used to anchor the CN catalyst, the main part of CO_2_ molecules is supplied directly from the gas phase with a high transport rate, thus a constant and higher interfacial CO_2_ concentration is maintained. As a result, the CO_2_ conversion rate and selectivity, as well as the amount of C2 molecules, are significantly higher over hydrophobic substrates than over hydrophilic substrates. To further demonstrate the significance of the continuous gas access, a diphasic system was analyzed as a reference, with the substrate immobilized with CN completely immersed in water (Figure 5 C). When the trapped CO_2_ molecules were isolated by the liquid phase, the diphase system disabled the continuous supply of CO_2_. Thus, the decreased concentration of interfacial CO_2_ resulted in lower conversion efficiency.

**Figure 5:**
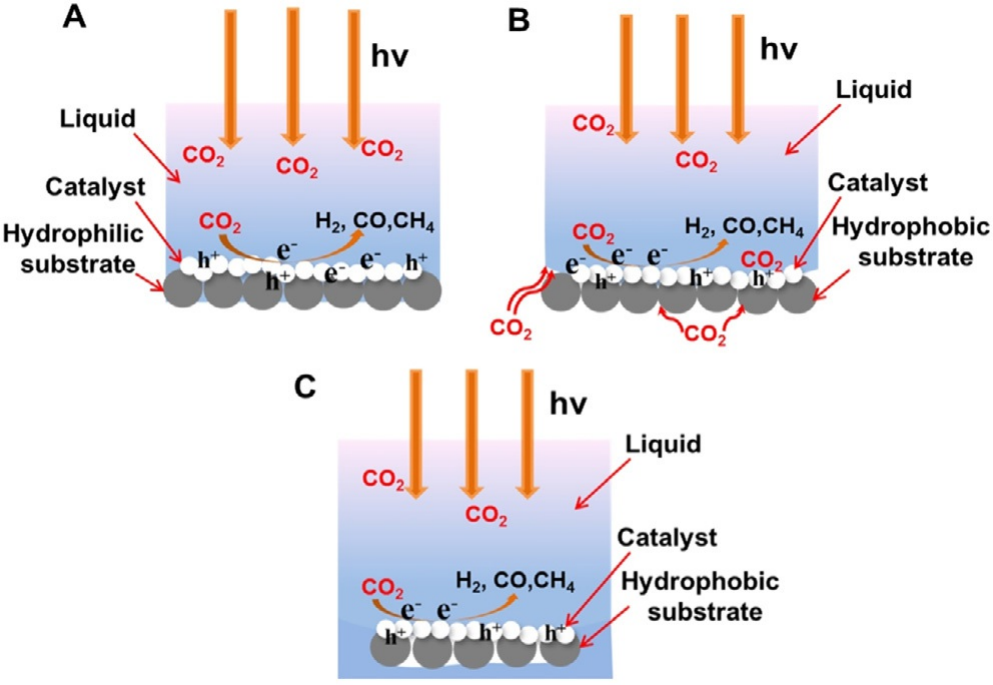
A, B) Behavior of photogenerated charge carriers and CO_2_ molecules for the hydrophilic substrate‐immobilized CN (A) and the hydrophobic substrate‐immobilized CN (B) in a triphase system. C) Schematic illustration of the CO_2_ molecule supply for the hydrophobic substrate‐immobilized CN that was completely immersed in the water.

There are still a number of means to improve the activity that were not explored in these first‐generation experiments. We still consider the rate‐determining step to be the four‐electron oxidation of water to dioxygen, which here proceeds in the absence of a cocatalyst. A kinetically much simpler reaction than O_2_ formation is the direct oxidation of phosphates to perphosphates.14a, 17 It was exciting to observe that CN/CF3 showed much higher activity when Na_3_PO_4_ was added to the water phase. In the triphase system, a CO_2_ conversion of 1413.85 μmol m^−2^ h^−1^ with a CO_2_ conversion selectivity of 95.5 % (Figure S7 and Table S2) and a quantum yield of 1.28 % were achieved. In this case, the synthetic photosynthesis even outperformed biological photosynthesis, in spite of its simplicity. Perphosphates therefore represent a valuable route of investigation, but also can be thermally or catalytically decomposed to liberate dioxygen to terminate product formation.^17^, ^18^

In summary, a triphase interfacial photocatalytic CO_2_ reduction system based on the gas–liquid–solid reaction interface allows efficient and continuous delivery of CO_2_ molecules to the catalyst surface and inhibits the hydrogen evolution reaction. The photogenerated charge carriers are efficiently utilized, resulting in significantly enhanced activity and selectivity in the photocatalytic CO_2_ reduction. In particular, the CN anchored onto the surface of a hydrophobic substrate (CN/CF3) exhibits about 7.2‐fold enhancement in the total CO_2_ conversion with 415.50 μmol m^−2^ h^−1^ as compared to the CN anchored onto the surface of superhydrophilic substrate (CN/CF1). This is accompanied by a CO_2_ conversion selectivity of 97.7 %, the remainder being the otherwise dominant H_2_ evolution. This product yield for photocatalytic CO_2_ reduction is also clearly superior to that with the conventional diphase system, and could be further enhanced by simplifying the photooxidation process from four‐electron dioxygen generation to perphosphate formation, with a CO_2_ conversion of 1413.85 μmol m^−2^ h^−1^ and a quantum yield of 1.28 %. Interestingly, a higher CO_2_‐based reactant flux also improved C_2_ hydrocarbon production, which reflects the higher chance of intermediate C_1_ species recombining at the catalyst surface. This work provides a platform to explore further interfacial architectures in system engineering of highly active semiconductor photocatalysts.

## Conflict of interest

*The authors declare no conflict of interest*.

## Supporting information

As a service to our authors and readers, this journal provides supporting information supplied by the authors. Such materials are peer reviewed and may be re‐organized for online delivery, but are not copy‐edited or typeset. Technical support issues arising from supporting information (other than missing files) should be addressed to the authors.

Supplementary
