# Supplementary material for: Improving Artificial Photosynthesis over Carbon Nitride by Gas–Liquid–Solid Interface Management for Full Light‐Induced CO2 Reduction to C1 and C2 Fuels and O2
Source: ChemSusChem. 2020 Feb 11;13(7):1730–4. doi: 10.1002/cssc.201903515 (PMC7187480; doi:10.1002/cssc.201903515)
Supplement: Supplementary file 1 — Supplementary [file CSSC-13-1730-s001.pdf]

CHEMISTRY & SUSTAINABILITY
CHEMSUSCHEM
ENERGY & MATERIALS

Supporting Information

Improving Artificial Photosynthesis over Carbon Nitride by Gas–Liquid–Solid Interface Management for Full Light-Induced CO_2 Reduction to C_1 and C_2 Fuels and O_2

Yang Xia,[a] Kai Xiao,[b] Bei Cheng,[a] Jiaguo Yu,*[a] Lei Jiang,[c] Markus Antonietti,[b] and Shaowen Cao*[a, b]

cssc_201903515_sm_miscellaneous_information.pdf

Experimental Procedures

Materials:

Section 1. Synthesis of polymeric carbon nitride (CN).

The carbon nitride used in this work was synthesized by a calcination method. In detail, 10 g of urea was placed in alumina crucibles with a cover. Then, the precursor was heated at 600 ^{o}C in air for 2 h with a heating rate of 5 ^{o}C min^{-1}. The resulting white product was collected for further use.

Section 2. Preparation of the immobilized photocatalyst.

Carbon fibers with/without polytetrafluoroethylene (PTFE) (hesen, 21cm × 20 cm) were used as the substrate to immobilize the polymeric carbon nitride (CN) nanosheets. Before the immobilization, the carbon fiber substrate was cleaned by sonication in water and ethanol, and then dried in air. The CN nanosheets were immobilized on the surface via a two-step method. Step 1: One side of the substrate was exposed to the ultraviolet irradiation machine (250 W, BZS250GF-TC) for several minutes to obtain the surface with different wettability. Step 2: A CN suspension (10 g/L) was sonicated for 6 h. The CN photocatalysts were immobilized on the substrate surface by dripping a 0.4 mL suspension forming an area about 1.0 cm^{-2}, and then dried at 70 °C for 3 h. Finally, the photocatalysts immobilized on different substrate were heated in a muffle furnace at 400 °C for 30 min and then allowed to cool to room temperature. Notably, the photocatalysts anchored on substrates with different wettability were denoted as CN/CF1, CN/CF2, and CN/CF3 (CN/CF1 and CN/CF2 represent the substrate without PTFE treated by the ultraviolet irradiation machine for 15 min and 2 min, respectively. CN/CF3 represents the substrate with PTFE treated by the ultraviolet irradiation machine for 30 min). For comparison, the CN/CF4 sample was prepared using the similar procedure with CN/CF3, except that the concentration of CN suspension was 20 g/L.

Characterization

The crystalline structures of the samples were probed by X-ray diffractometer with Cu Kα radiation (Rigaku, Japan). The morphology was examined by a field emission scanning electron microscope (FESEM, JSM-7500). UV–vis diffuse reflectance spectra (DRS) of the samples were obtained on a UV–vis spectrometer (UV-2600, Shimadzu). Photoluminescence (PL) emission spectra were recorded at room temperature using a Fluorescence Spectrophotometer (F-7000, Hitachi, Japan) with a 315 nm excitation wavelength. The Fourier transform infrared spectra (FTIR) were acquired using a Thermo Fisher Nicolet iS50 FT-IR spectrometer. A contact angle (CA) goniometer (Theta Flex, Biolin Scientific, Finland) was

used to measure the water CA.

Photocatalytic CO_2 reduction measurements

All of the samples were degassed at 150 ^{o}C for 6 h prior to the measurements. Specifically, a two-neck 200 mL customized glass flask was used as reactor. In the triphase system, three pieces of substrate immobilizing with CN leaned against the inner wall of reactor; the portion of substrate immobilized with CN was immersed in water (55 mL), while another part of substrate (free of CN) was exposed to gas atmosphere. 0.084 g of $NaHCO_3$ was added to the neck of reactor, and N_2 was blown through the reactor to ensure anaerobic conditions before light irradiation. H_2SO_4 aqueous solution (0.3 mL, 2 M) was syringed into the reaction system to produce CO_2 sources. A 300 W Xe lamp with an AM1.5 filter (100 mW/cm^2) was employed as the light source. The resulting products were detected by a gas chromatography (GC-2014C, Shimadzu, JAPAN). For comparison, the photocatalytic CO_2 reduction in diphase system was performed with the same condition of triphase system, except that the substrate immobilizing with CN was completely immersed in water (75 mL). To investigate the stability of the sample on CO_2 conversion, the sample was collected after each photocatalytic reduction reaction and recycled for 4 runs with the same procedure.

Phosphate-promoting experiment for CO_2 photoreduction was carried out similarly to the above-mentioned experiments, except changing the water to 0.1 M Na_3PO_4 solution.

Isotopic measurement

The experimental process of isotopic measurement is same with that of photocatalytic CO_2-reduction experiments, except that NaH(^{12}C)O_3 is replaced by NaH(^{13}C)O_3 (Cambridge Isotope Laboratories Inc., USA), and H_2O is replaced by D_2O (Cambridge Isotope Laboratories Inc., USA). Then, $^{12}CH_4$, $^{13}CH_4$, ^{12}CO, ^{13}CO, $^{13}C_2H_4$ and $^{13}C_2D_4$ species were detected by a gas chromatography-mass spectrometer (Agilent Technologies 6890N GC system with 5975 MSD, USA).

Calculation of selectivity

The selectivity of photocatalytic CO_2 reduction to valuable fuels was calculated using the following equation:

$$Selectivity\ of\ \text{valuable hydrocarbons (\%)} = \frac{[8n(CH_4)+2n(CO)+12n(C_2H_4)]}{[8n(CH_4)+2n(CO)+12n(C_2H_4)+2n(H_2)]}\times 100\%$$

Where n(CO), n(CH_4) n(C_2H_4)and n(H_2) are the amounts (moles) of CO, CH_4, C_2H_4 and H_2 formed within a certain period of time.

Quantum Yield Calculation

The photochemical quantum yield (Φ) is an important parameter to characterize the photoreduction performance. The light intensity in the effective range (280 to 450 nm) was estimated from the measured light spectrum of the 300 W Xe lamp with an AM1.5 filter in this work (Figure S8) using the following equation[1]:

$$P = \int_{\lambda_m}^{\lambda_n} f(\lambda)d\lambda = \sum_{i=m}^{i=n}\left(\frac{A_m + A_n}{2}\right) \bullet \Delta\lambda$$

Where λ is the wavelength (nm), A_m and A_n provide the simulated solar spectral irradiance (μW m^{-2} nm^{-1}) in the wavelength of λ_m and λ_n. Thus, the average wavelength in the effective range (280 to 450 nm) was estimated to be 329 nm.

The photon energy at a certain wavelength is calculated using the following equation:

$$E_{photon} = \frac{hc}{\lambda}$$

Where h is the Plank constant, c is the speed of light, and λ is the wavelength. The average photon energy is estimated by using the average wavelength in the range of 280 to 450 nm. The deposited catalyst film area (S) is 3 cm^2 (three pieces of 1 cm^2 catalyst film on the substrate).

The mole of photo absorbed by catalyst (n) is calculated using the following equation:

$$n = \frac{P \times S \times t}{N_A \times E_{photon}}$$

Where P is the light intensity, S is the catalyst film area, t is the irradiation time, N_A is the Avogadro's constant. Therefore, quantum yields of CO, CH_4, and C_2H_4 can be calculated using the following equations[2,3]. Two, eight, twelve electrons are required to convert CO_2 to CO, CH_4, and C_2H_4, respectively.

$$\Phi_{co}(\%) = \frac{2\ mol\ of\ CO\ yield}{moles\ of\ photon\ absorbed\ by\ catalyst} \times 100\% \quad (1)$$

$$\Phi_{CH_4}(\%) = \frac{8\ mol\ of\ CH_4\ yield}{moles\ of\ photon\ absorbed\ by\ catalyst} \times 100\% \quad (2)$$

$$\Phi_{C_2H_4}(\%) = \frac{12\ mol\ of\ C_2H_4\ yield}{moles\ of\ photon\ absorbed\ by\ catalyst} \times 100\% \quad (3)$$

Table S1 The yield of gas products (μmol m^{-2} h^{-1}) of triphase CO_2 photoreduction system over various photocatalysts, without any sacrificial agent.

Samples	CH_4	CO	C_2H_4	Total CO_2 conversion[a]	H_2	CO_2 photoreduction selectivity (%)
CN/CF1	9.26	41.25	3.54	57.59	33.81	74.6
CN/CF2	15.91	73.47	10.9	111.18	28.24	87.8
CN/CF3	47.16	132.34	118	415.50	23.70	97.7
CN/CF4	79.23	210.12	189.7	668.75	50.44	97.1

[a]Total CO_2 conversion = n(CH_4) + n(CO) + 2n(C_2H_4)

Table S2 The yield of gas products (μmol m^{-2} h^{-1}) of CO_2 photoreduction over CN/CF3 in the presence of 0.1 M Na_3PO_4 solution

Samples	CH_4	CO	C_2H_4	Total CO_2 conversion[a]	H_2	CO_2 photoreduction selectivity (%)
triphase	303.32	284.41	413.06	1413.85	185.84	95.5
diphase	182.07	154.48	317.11	970.77	283.49	90.7

[a]Total CO_2 conversion = n(CH_4) + n(CO) + 2n(C_2H_4)

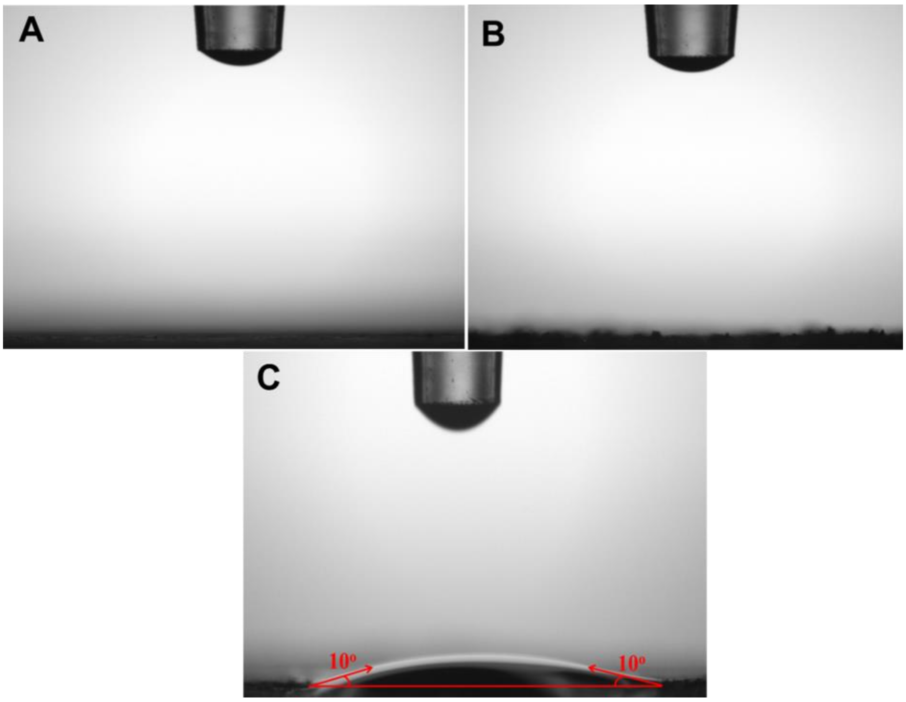

Figure S1. Contact angle measurement photographs of water droplet that placed on the CN immobilized substrates with different wettability: (A) CN/CF1, (B) CN/CF2 and (C) CN/CF3.

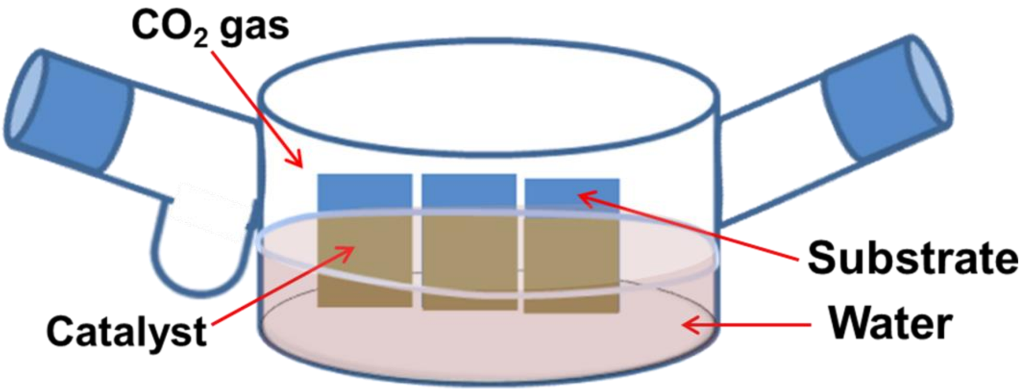

Figure S2. Experimental setup for the triphase photocatalytic reaction. The portion of substrate immobilizing with CN was immersed in water, while another part of substrate (free of CN) was exposed to CO_2 gas. The availability of CO_2 at the reaction interface is dependent upon its mass transfer mainly through the substrate.

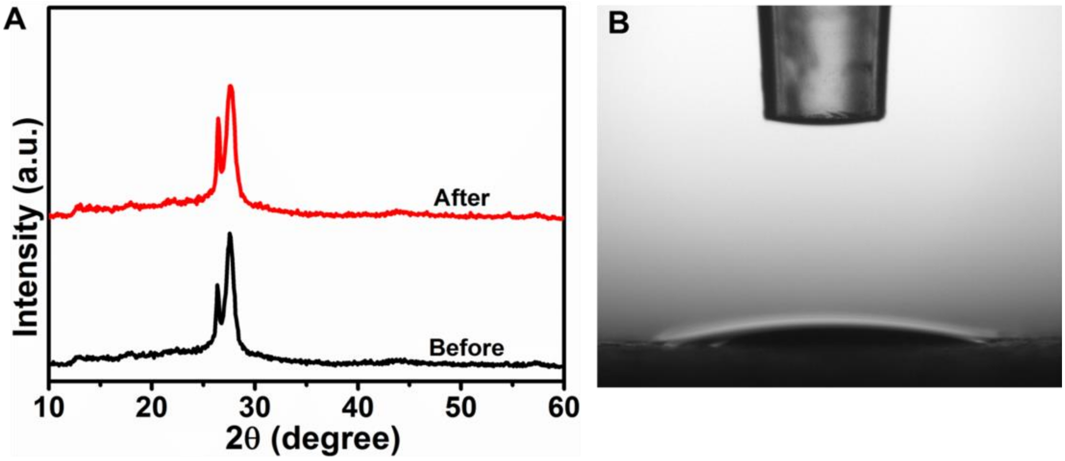

Figure S3. (A) XRD patterns of CN/CF3 before and after the stability measurement in the triphase system. (B) Contact angle measurement photographs of water droplet over CN/CF3 after the stability measurement in the triphase system.

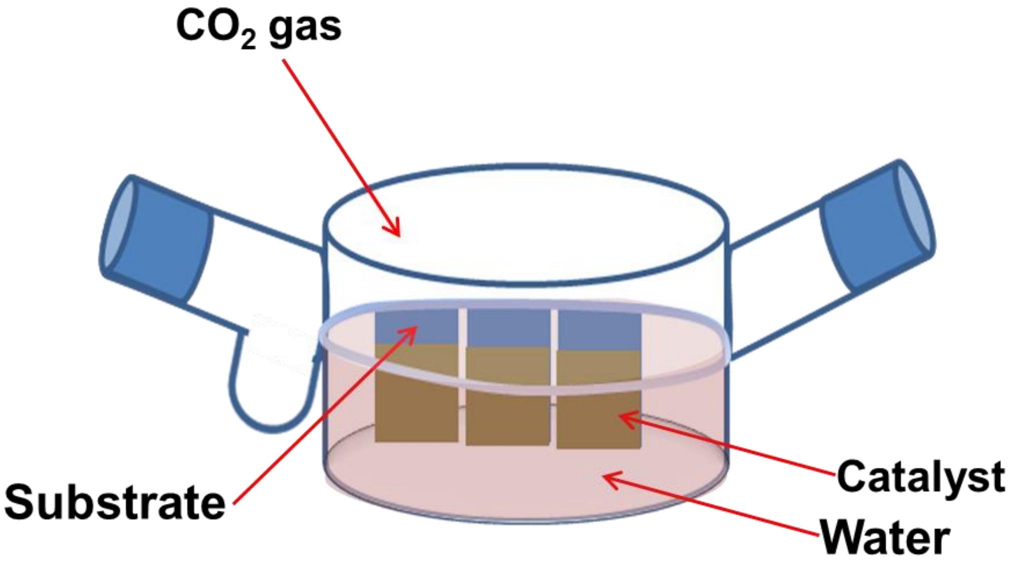

Figure S4. Experimental setup for the diphase photocatalytic reaction. The substrate immobilizing with CN was completely immersed in water, while the availability of CO_2 at the reaction interface is dependent upon its mass transfer through the water phase.

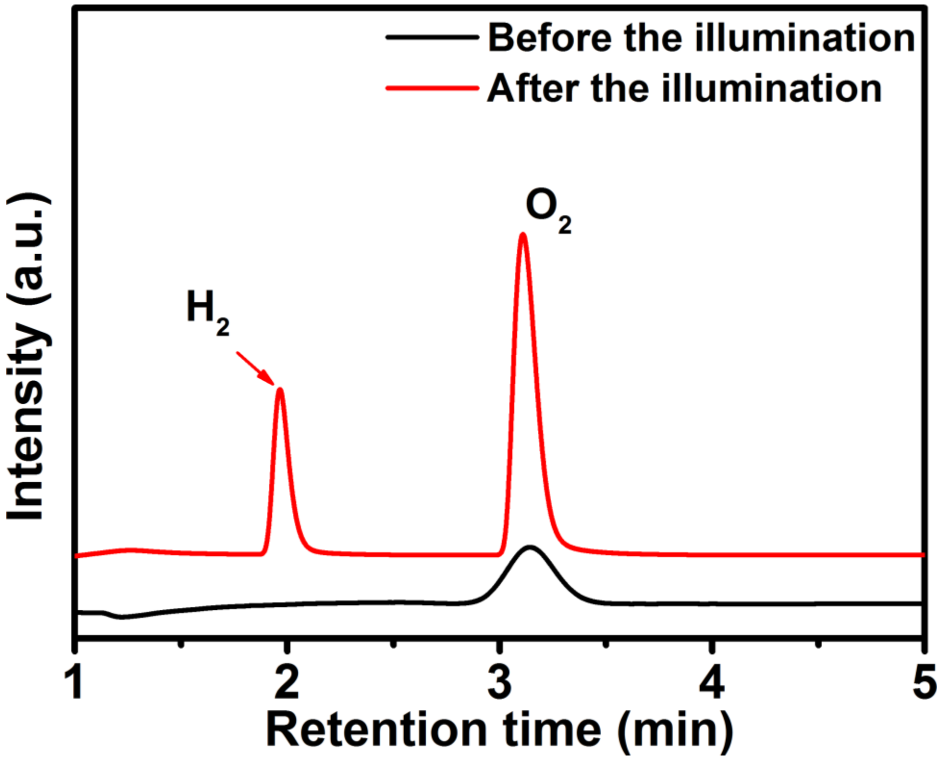

Figure S5. The GC spectra of resulting H_2 and O_2 after photocatalytic CO_2 reduction reaction over CN/CF3 in triphase system.

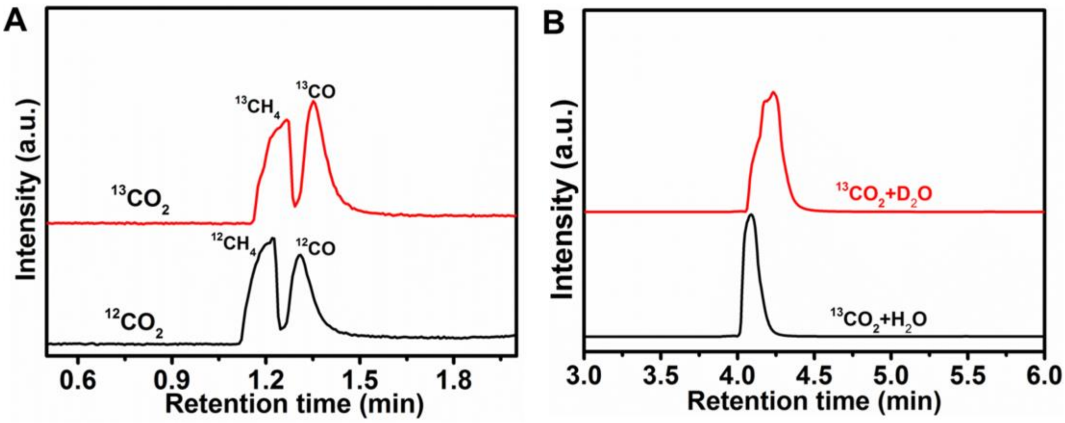

Figure S6. (A) GC-MS spectra over CN/CF3 in triphase system after irradiation for several hours with $^{12}CO_2$ and $^{13}CO_2$ as carbon sources. (B) GC-MS spectra over CN/CF3 in triphase system after irradiation for several hours with $^{13}CO_2$ as carbon source, D_2O and H_2O as hydrogen sources, respectively.

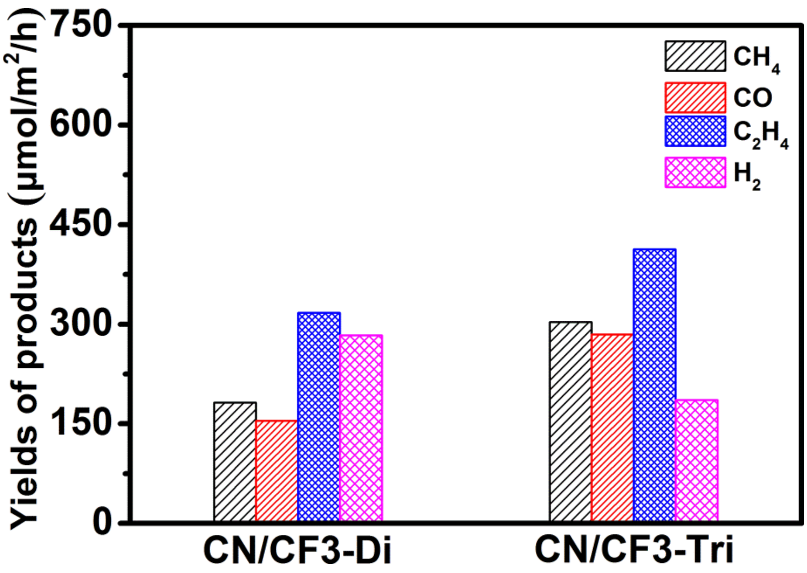

Figure S7. Comparison of photocatalytic activity over CN/CF3 in the presence of 0.1 M Na_3PO_4 solution in the diphase and triphase systems.

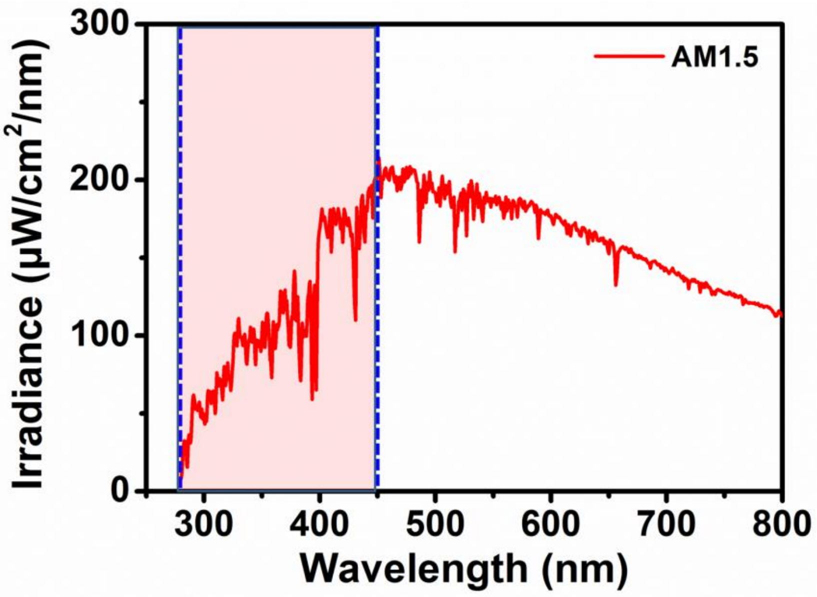

Figure S8. Light spectrum of the 300 W Xe lamp with an AM1.5 filter. The spectrum was measured at room temperature and at the distance of 5 cm away from the light soure, which was the exact distance that the catalyst was immobilized.

[1] W. Wang, W. An, B. Ramalingam, S. Mukherjee, D. Niedzwiedzki, S. Gangopadhyay, P. Biswas, *J. Am. Chem. Soc.* **2012,** 134, 11276–11281.

[2] Y. Li, W. Wang, Z. Zhang, M. Woo, C. Wu, P. Biswas, *Appl. Catal. B.-Envrion.* **2010,** 100, 386–392.

[3] J. Low, L. Zhang, T. Tong, B. Shen, J. Yu, *J. Catal.* **2018,** 361, 255–266.
